# Cryptography in Hierarchical Coded Caching: System Model and Cost Analysis

**DOI:** 10.3390/e23111459

**Published:** 2021-11-03

**Authors:** Behrouz Zolfaghari, Vikrant Singh, Brijesh Kumar Rai, Khodakhast Bibak, Takeshi Koshiba

**Affiliations:** 1 CSE Department, Indian Institute of Technology Guwahati, Guwahati 781039, Assam, India; behrouz@cybersciencelab.org; 2 Cyber Science Lab, School of Computer Science, University of Guelph, Guelph, ON N1G 2W1, Canada; 3 EEE Department, Indian Institute of Technology Guwahati, Guwahati 781039, Assam, India; vs13@iitg.ac.in (V.S.); brijesh.rai@gmail.com (B.K.R.); 4 Department of Computer Science and Software Engineering, Miami University, Oxford, OH 45056, USA; 5 Department of Mathematics, Faculty of Education and Integrated Arts and Sciences, Waseda University, Tokyo 169-8050, Japan; tkoshiba@waseda.jp

**Keywords:** coded caching, secure delivery, hierarchical coded caching, cost analysis, system model

## Abstract

The idea behind network caching is to reduce network traffic during peak hours via transmitting frequently-requested content items to end users during off-peak hours. However, due to limited cache sizes and unpredictable access patterns, this might not totally eliminate the need for data transmission during peak hours. Coded caching was introduced to further reduce the peak hour traffic. The idea of coded caching is based on sending coded content which can be decoded in different ways by different users. This allows the server to service multiple requests by transmitting a single content item. Research works regarding coded caching traditionally adopt a simple network topology consisting of a single server, a single hub, a shared link connecting the server to the hub, and private links which connect the users to the hub. Building on the results of Sengupta et al. (IEEE Trans. Inf. Forensics Secur., 2015), we propose and evaluate a yet more complex system model that takes into consideration both throughput and security via combining the mentioned ideas. It is demonstrated that the achievable rates in the proposed model are within a constant multiplicative and additive gap with the minimum secure rates.

## 1. Introduction

Coded caching, proposed by Maddah-Ali and Niesen [1], refers to an augmented variant of caching. Coded caching follows two strategies during two transmission phases in order to avoid a traffic bottleneck in the network. The first transmission phase, referred to as the placement phase, takes place in off-peak hours. During this phase the system attempts at placing frequently-demanded content items in local memories of corresponding interested users in order to avoid unnecessary transmission during peak time. This helps deteriorate network bandwidth over utilization and underutilization problems during peak and off-peak intervals. An effective placement strategy should consider the statistical and probabilistic nature of the users’ access patterns. The second phase, i.e., the delivery phase manages the transmission in peak hours. The ideal goal in the latter phase is to send only a single coded content item which is a function of the originally-requested content items. Each user—in the ideal case—should be able to calculate its own demanded item from the transmitted item. The more the system approaches this goal, the less amount of transmission during the delivery phase (rate) is required.

The authors in [1] made a lot of simplifying assumptions when establishing the first system model for a coded caching scheme. They assumed a simple network based on a star topology which provides a one-way content transmission from a single server storing *N* files each of size *F* bits to *K* users and each user having cache size of MF bits. Each user requests a single file during the delivery phase. Every file sent by the server passes through a single shared link and arrives at the hub where it is duplicated and transmitted to each user through a private link.

This system model is obviously not realistic enough because it ignores many considerations of a real-world network among which we focus on scalability and security in this paper. There are various security-related issues, such as confidentiality, privacy, and distributed denial-of-service (DDoS) attack protection which need to be addressed in coded caching. Among the mentioned issues, confidentiality has received the most focus in recent years [2,3]. Previous works in this area have augmented the coded caching system model by adding an adversary with access to the shared link only during the delivery phase. The space required to store the cryptographic keys in the server memory and user caches, as well as the extra traffic caused by key exchange mechanisms should be considered as obvious costs of this variant of coded caching.

In order to address the scalability issue, some researchers have augmented the coded caching system model in another way via proposing hierarchical network topology [4]. In the proposed topology, the main server is mirrored in each cluster of users. This allows part of the traffic to be locally handled in user clusters which leads to improved scalability. This improvement is achieved at the cost of redundant servers and links.

Although scalability and security have been separately examined in previous research, the literature in this area has not come up with a study on the possibility or the costs of considering both issues at the same time. This paper addresses both of the mentioned issues via considering confidential content transmission over a hierarchical network. This goal is achieved by further augmenting the coded caching system model, as well as analyzing the related costs. In our proposed system model, the adversary can eavesdrop the shared links in each hierarchy level during the peak interval.

The costs of scalability have already been analyzed in previous research [4]. We compare the results of our mathematical cost evaluations with those obtained in [4] to analyze the extra cost posed by confidentiality considerations. The key contribution of the paper is the result that although the achievable rates are within a constant multiplicative and additive gap to the corresponding lower bounds in both schemes, confidentiality causes the constants to grow larger.

The rest of this paper is organized as follows. Section 2 defines the problem we are tackling in this paper. This section first studies relevant works and presents some preliminaries, and then the shortcomings of the previous works which motivate our work in this paper are discussed. Section 3 explains the secure hierarchical coded caching scheme and describes the system model and configuration. The fundamental limits as well as costs are analyzed in Section 4. In this section, the secure achievable rates, memory requirements and the lower bounds on the rates are calculated. A gap analysis between the secure achievable rates and the corresponding lower bounds is presented in Section 5. The last section of this paper is Section 6 which concludes the paper and suggests further research topics.

## 2. Problem Statement

In this section, we first present some preliminary discussions regarding coded caching and review the related literature and then highlight some shortcomings in the related works which motivate us to propose the secure hierarchical coded caching scheme.

### 2.1. Related Works

Caching is a solution to the problem of temporally-nonuniform access to contents stored in servers which may causes the network bandwidth to be underutilized in the off-peak interval while it can render a bottleneck in the peak interval. This technique helps achieve more uniform network traffic and deteriorate the bottleneck problem by allowing the system to store frequently-accessed content items in local caches during the off-peak time.

Coded caching has been a research focus during recent years [5,6,7,8,9]. Coded caching is finding its application in modern technologies and services, such as content delivery [10,11,12], mobile computing [13,14,15], and information-enteric networks [16]. Different aspects of coded caching have recently been studied among which we can refer to as [17], centralized [18,19] and decentralized [18,19] coded caching, placement [20] and delivery [21,22] schemes, as well as added pre-fetching phase [23], multi-casting [22,24,25,26], scheduling [27], error correction [28], clustering [29], heterogeneity [12,25,30], the impact of file size [31,32], dealing with non-uniform user demands [33] and peak-time traffic reduction [1]. Moreover, security in coded caching has been considered as a concern [20,34,35,36] and cryptography has been among the best-studied security mechanisms for use in coded caching [37,38].

Examining the above problems has led to different variants of caching schemes. In this paper, we focus on coded caching schemes which try to service as many user requests as possible by transmitting a single coded data item in the peak time. Coded caching schemes can be classified into the following categories with respect to their behaviors in the placement phase.

### 2.2. Centralized Schemes

In these schemes, the server decides the data items which are to be stored in user caches during the placement phase [1,39,40,41,42,43]. It has been shown that a multiplicative factor of $\frac{1}{1+KM/N}$ in size reduces the rate in centralized coded caching. This factor is referred to as *global caching gain*. As shown in [1], the centralized coded caching rate $R_{C}\left(M\right)$ is given by (1),

(1) $$R_{C}\left(M\right)≜K\cdot \left(1-M/N\right)\cdot \min\left\{\frac{1}{1+KM/N},\frac{N}{K}\right\}.$$

### 2.3. Decentralized Schemes

In the latter schemes, users are allowed to store random data in their caches. It was shown in [44] that the rate [1] in decentralized coded caching can be obtained from (2),

(2) $$R_{D}\left(M\right)≜K\cdot \left(1-M/N\right)\cdot \min\left\{\frac{N}{KM}\left(1-{\left(1-M/N\right)}^{K}\right),\frac{N}{K}\right\}.$$

An important point to note here is that the term “decentralized” does not refer to the underlying network and the network topology adopted in [44,45] are the same as the one considered in [1].

### 2.4. Hierarchical Coded Caching Scheme

The scheme introduced in [4] proposes a hierarchical coded caching scheme in which the content stored in the main server can be mirrored by intermediate servers in different levels of hierarchy before being placed in end user caches. In this scheme, the requests issued by each end user are first forwarded to the closest intermediate server. If not serviced the request is then forwarded to the higher hierarchy level. This implies the existence of different peak and off-peak intervals in different hierarchy levels.

Two different caching schemes have been proposed in this paper. The first scheme referred to as Scheme A allows simultaneous coded multicasting in both hierarchy levels. Each mirror first downloads the content items requested by its corresponding users from the main servers. Then, the items are coded and forwarded to the users. In Scheme B, mirrors act as memory-less routers. They receive the items from the main server and forward them without being stored or coded. It has been demonstrated that both schemes can individually perform sub-optimally [4]. The authors in this paper argued that because of the disjunctive relation between Scheme A and Scheme B, the rate of each link is the sum of the individual rates induced by the two schemes. They proposed a hybrid scheme named as the *generalized coded caching scheme* that attempts to incorporates a proper combination of Scheme A and Scheme B in order to approximately minimize the overall rate.

### 2.5. Secure Coded Caching Scheme

The scheme presented in [3] argued that the shared link may be eavesdropped by an adversary since it is publicly accessible as a broadcast medium. Thus they proposed an on time pad (OTP) cryptosystem [46] to preserve the confidentiality of the data items exchanged through this link. In their proposed scheme, the keys are placed in user cache along with the data in the placement phase. These confidentiality measures can be applied in both centralized and decentralized coded caching systems.

It is demonstrated in this paper that the secure rates for the centralized scheme and the decentralized scheme can be obtained through replacing M/N by (M−1)/(N−1) in Equations (1) and (2), respectively. The authors of [4] argued that the overall rate of the hierarchical network is the sum of the individual rates in different levels of the hierarchy. Thus, if the overall rate needs to be minimized, both levels should operate at their minimum rates.

The relationship between the goals followed by the mentioned schemes motivates our work in this paper. Moreover, we compare our results with the ones obtained in [4] as reference.

### 2.6. Motivations

The researchers who proposed the idea of coded caching made several simplifying assumptions regarding the system model [1]. These assumptions made the core idea more manageable in its early days. However, several aspects of the primary system model obviously need to be revisited in order for the scheme to be applicable to real-world networks. Scalability and security are two aspects considered by other researchers [3,4]. However, there are still several related issues which can motivate further research. For example, It should be considered that confidentiality (addressed in [3]) is not the only aspect of security. Moreover, the network topology (studied in [4]) is not the only factor affecting scalability. However, what motivates us for the work of this paper is the lack of a research on a system model which is both secure and confidential.

Achieving the confidentiality promised in [3], as well as the scalability of the network studied in [4] by combining both ideas looks an enticing natural idea. However, the important issue to consider here is that combining these ideas can bring about new problems. In fact, the traffic and memory space overhead caused by the secure coded caching is against the scalability aimed by the hierarchical network. The key transmission occupies the bandwidth of the network which adversely affects the scalability. This problem will look more prominent when we consider the fact that OTP requires a new key for each single transmission. On the other hand, storing the keys in user caches prevents some frequently-requested data items to be stored during the placement phase because of the limited cache sizes. This will affect the peak time rate and may, consequently, overshadow the scalability of the underlying hierarchical network. Thus, every research focusing on simultaneous confidentiality and scalability should consider the trade-off between the two parameters. This trade-off will appear as an extra cost induced by the security-related constraints which should be tolerated by the hierarchical coded caching scheme.

In this paper, we first present an extended coded caching scheme which incorporates OTP confidentiality provisions and hierarchical network topology in the system model. Then, we analyze the extra cost induced by confidentiality via comparing the rate bounds to the case of non-secure hierarchical coded caching.

## 3. Secure Hierarchical Coded Caching

In this section, we present our secure hierarchical coded caching scheme and the related system model. Our system model needs to be defined in two aspects. We first introduce the topology and resources of the underlying network and then discuss the caching scheme which describes the transmissions in placement and delivery phases. Next, we discuss the security-related considerations.

In terms of topology, we adopt the 2-level hierarchical topology, described in [4]. In the top level of the hierarchy, the main server is connected to a hub via a shared link and then to mirror servers via separate links. In each cluster in the next hierarchy level, a shared link connects the mirror server to the hub while end users are connected to the same hub using separate links. We assume the number of the clusters to be equal to $K_{1}$ each of which connects $K_{2}$ end users.

As for the resources, the main server is assumed to store *N* files represented by $W_{1}$ through $W_{N}$ each of size *F* bits. We assumed that the bits in a file are independent and uniformly distributed. The cache sizes in the mirrors and the end users are assumed to be $M_{1}F$ and $M_{2}F$ bits, respectively. The main and mirror servers are assumed to have unlimited processing power.

With respect to the caching scheme, we will assume the generalized caching scheme presented in [4] which is a combination of Scheme A and Scheme B. We follow the procedure to find the most efficient combination of the schemes.

During the delivery phase, each user makes exactly one demand. The local demands in each cluster are collected by the corresponding mirror server and then forwarded to the main server. The demand issued by $U_{\left(i,j\right)}$ is represented by the element $d_{i,j}$ in the demand matrix D. According to the demands, the main server encodes the proper content along with with the orthogonal keys and transmits them within a file $X^{D}$ of size $R_{S_{1}}F$ bits to all mirrors. Then, each mirror re-encodes (Scheme A) or forwards (Scheme B) the data requested by its corresponding users along with the related keys and transmits them within a file $Y^{D}$ of size $R_{S_{2}}F$ bits.

**Figure 1:**
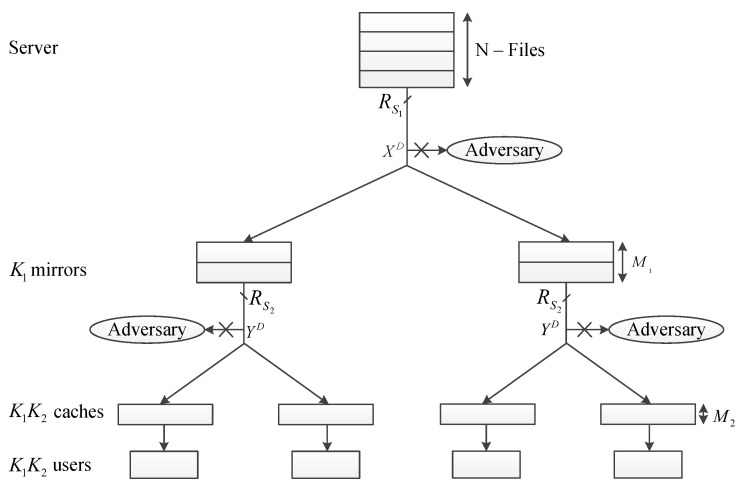
A hierarchical caching system with external adversaries acting overall shared links.

Security-related constraints are considered in order to keep the transferred contents confidential from an external adversary assumed to have access to every shared link. In order to add confidentiality to our caching scheme, we adopt the security constraints proposed in [2,3]. Adopting the orthogonal key scheme proposed in [3], user caches, as well as mirror server memories are considered to be partitioned into Data and Key regions in order to keep space for storing the keys in the placement phase. Figure 1 shows the access model of adversary as well as the security-related configuration.

The mentioned security constraints guarantees that $I\left(X^{D};W_{1},W_{2},\ldots ,W_{N}\right)=\epsilon_{1}$ and $I(Y^{D};W_{1},$$W_{2},\ldots ,W_{N})=\epsilon_{2}$, where $\epsilon_{1}\to 0$ and $\epsilon_{2}\to 0$ which states that the external adversary cannot reveal any information regarding the files $W_{1},W_{2},\ldots ,W_{N}$ by eavesdropping the shared links without access to users’ and mirrors’ caches. It is to be noted that $\epsilon_{1}\to 0$ and $\epsilon_{2}\to 0$ are for the case when file size is sufficiently large, i.e., when file size →∞. The minimum number of mirrors or users needed to be compromised in order to break the security was discussed in [3].

Adopting the security constraints from [3] requires some extra assumption regarding the placement phase in Scheme A. Since the users cannot establish an immediate communication with the main server, we assume a delegation between the main server and mirror servers in the placement phase in Scheme A. It means that the mirror servers are trusted and granted access to the keys because they need to decrypt and encrypt the contents again before and after re-encoding them.

Another assumption adopted from [3] in our system model is that every user is interested in no more than one file in the delivery phase and the demanded files are mutually different. The system cannot allocate resources, such as private links, network bandwidth, and cache space to a user with no demands in the delivery phase. Therefore, we suppose every user makes exactly one request in this phase. The latter assumptions obviously result in $N\geq K_{1}K_{2}$ as a criterion for the server to be able to answer all user requests. Again, we note that it is not reasonable to store files which will never be demanded. Thus, we assume that $N=K_{1}K_{2}$. Throughout, we assume that the placement phase is secure and links are error-free.

Let us represent the secure rate in the top hierarchy level by $R_{S_{1}}$ and the second level secure rate by $R_{S_{2}}$. For a demand matrix D and for a large-enough file size *F*, a tuple $\left(M_{1},M_{2},R_{S_{1}},R_{S_{2}}\right)$ is said to be *feasible for D* if each user $U_{\left(i,j\right)}$ is able to recover its requested file $d_{i,j}$ securely with a probability arbitrarily close to unity. Moreover, $\left(M_{1},M_{2},R_{S_{1}},R_{S_{2}}\right)$ is *feasible* if it is feasible for all possible request matrices D. Throughout, we assume feasible rate region in our analysis.

## 4. Fundamental Limits and Cost Analysis

The procedure we follow in our evaluations in this section can be described as follows. In order to maximize the secure achievable rate in the generalized scheme, we try to find the most effective combination of the Schemes A and B. To do this, we first parameterize the combination. We assume that a fraction equal to α of each file residing in the server (as well as transmissions in the top hierarchy level) are ruled by Scheme A and the rest (1−α) are transmitted on the basis of Scheme B. The corresponding fractions in the user cache (as well as transmissions in the second hierarchy level) are assumed to be equal to β and 1−β, respectively. Then we try to find the best possible values for α and β which will result in the most effective combination. We denote the latter values by $\alpha^{*}$ and $\beta^{*}$. In the next step, we calculate the secure achievable rate for the generalized scheme via calculating the rates for both Schemes A and B and then combining the results together assigning the values $\alpha^{*}$ and $\beta^{*}$ to α and β. We calculate the lower bounds of the rates through a similar procedure and then analyze the gap between the achievable rates and the rates specified by the lower bounds. A comparison between our results and those obtained in [4] highlights the cost of security in hierarchical network caching.

### 4.1. Preliminary Discussions

While analyzing the rates in each scheme, we separately consider each of the three regimes proposed in [4]. This makes it plausible to compare our results to those obtained in [4]. The mentioned regimes are characterized as shown in Equation (3) in terms of $M_{1}$ and $M_{2}$,

(3) $$\begin{array}{ccc} 1M_{1}+M_{2}K_{2} & \geq & N\;\;\mathrm{and}\;\;0\leq M_{1}\leq N/4,\\ 2M_{1}+M_{2}K_{2} & < & N,\\ 3M_{1}+M_{2}K_{2} & \geq & N\;\;\mathrm{and}\;\;N/4<M_{1}\leq N. \end{array}$$

The results in [4], to which we compare our own results are as follows. The optimum values of α and β for the mentioned regimes in the non-secure hierarchical coded caching scheme are [4],

(4) $$\begin{array}{c} \left(\alpha^{*},\beta^{*}\right)≜\left\{\begin{array}{cc} \;\left(\frac{M_{1}}{N},\frac{M_{1}}{N}\right) & \mathrm{in}\;\mathrm{regime}\;1,\\ \;\left(\frac{M_{1}}{M_{1}+M_{2}K_{2}},0\right) & \mathrm{in}\;\mathrm{regime}\;2,\\ \left(\frac{M_{1}}{N},\frac{1}{4}\right) & \mathrm{in}\;\mathrm{regime}\;3. \end{array}\right. \end{array}$$

Moreover, the corresponding non-secure achievable rates for Scheme A and Scheme B have been calculated as functions of $\alpha^{*}$ and $\beta^{*}$ in [4],

(5) $$R_{1}\left(\alpha^{*},\beta^{*}\right)\approx \left\{\begin{array}{cc} \min\left\{\frac{K_{1}K_{2}}{1},\frac{N}{M_{2}}\right\} & \mathrm{in}\;\mathrm{regime}\;1,\;\\ \min\left\{K_{1}K_{2},\;\frac{M_{1}}{M_{1}+M_{2}K_{2}}\cdot \frac{\left(N-M_{1}\right)K_{2}}{M_{1}+M_{2}K_{2}},\;+\frac{M_{1}}{M_{1}+M_{2}K_{2}}\cdot \frac{NK_{2}-M_{1}}{M_{1}+M_{2}K_{2}}\right\} & \mathrm{in}\;\mathrm{regime}\;2\;,\\ \frac{{\left(N-M_{1}\right)}^{2}}{NM_{2}} & \mathrm{in}\;\mathrm{regime}\;3, \end{array}\right.$$

(6) $$R_{2}\left(\alpha^{*},\beta^{*}\right)\approx \min\left\{K_{2},\frac{N}{M_{2}}\right\}.$$

**Figure 2:**
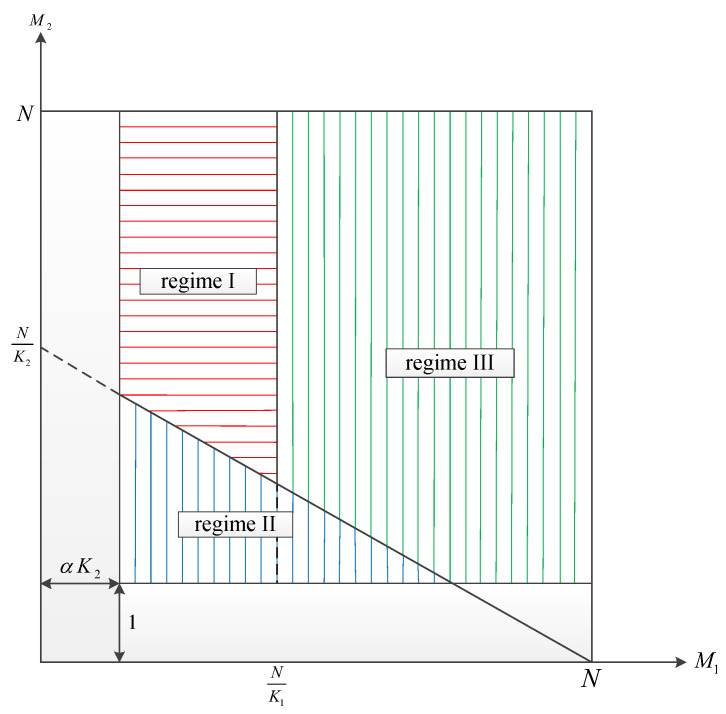
Different regimes of $M_{1},\;M_{2}$ for $\alpha^{*}$ and $\beta^{*}$.

See Figure 2 for different regimes of $M_{1},\;M_{2}$ for $\alpha^{*}$ and $\beta^{*}$. In (4) and (5), the approximation is within a constant additive and multiplicative as given by (7) and (8),

(7) $$\begin{array}{ccc} R_{1} & \geq & R_{1}^{lb}\left(M_{1},M_{2}\right)\geq \frac{1}{60}R_{1}\left(\alpha^{*},\beta^{*}\right)-4, \end{array}$$

(8) $$\begin{array}{ccc} R_{2} & \geq & R_{2}^{lb}\left(M_{1},M_{2}\right)\geq \frac{1}{36}R_{1}\left(\alpha^{*},\beta^{*}\right)-16. \end{array}$$

### 4.2. Secure Achievable Rates

Before beginning to develop our mathematical modelings, let us introduce some notations we will use in our equations. We will refer to the *j*th user ($j\in [1,2,\ldots ,K_{2}]$ ) in the *i*th cluster where $i\in [1,2,\ldots ,K_{1}]$ as $U_{\left(i,j\right)}$ and refer to the corresponding cache as $C_{\left(i,j\right)}$. Let us represent the coded content items transmitted in the first and second levels of the hierarchy by $X^{D}$ and $Y^{D}$, respectively, where D is the request matrix. Furthermore, let us represent the secure rate in the top hierarchy level by $R_{S_{1}}$ and the second level secure rate by $R_{S_{2}}$.

Now, let us begin the derivation of our model by calculating $R_{S_{1}}$ and $R_{S_{2}}$ for scheme *A*. For *N* files and $K_{1}$ mirrors each with a cache size of $M_{1}\in \frac{N-K_{2}}{K_{1}}\cdot t_{1}+K_{2}$, where $t_{1}\in \left\{0,1,2,\cdots ,K_{1}\right\}$, $R_{S_{1}}$ for scheme A is given by

(9) $$R_{S_{1}}^{A}=K_{2}\cdot r\left(\frac{M_{1}-K_{2}}{N-K_{2}},K_{1}\right),$$

where r(.,.) is defined as:

(10) $$\begin{array}{ccc} r\left(\frac{M}{N},K\right) & ≜ & {\left[K\cdot \left(1-\frac{M}{N}\right)\cdot \frac{N}{KM}\left(1-{\left(1-\frac{M}{N}\right)}^{K}\right)\right]}^{+}, \end{array}$$

with ${\left[x\right]}^{+}≜max\left\{x,0\right\}$. Moreover, $R_{S_{2}}$ for Scheme A considering $K_{2}$ users each with a cache size of $M_{2}\in \frac{N-1}{K_{2}}\cdot t_{2}+1$, where $t_{2}\in \left\{0,1,\ldots ,K_{2}\right\}$, can be obtained from

(11) $$R_{S_{2}}^{A}=r\left(\frac{M_{2}-1}{N-1},K_{2}\right).$$

Similarly, $R_{S_{1}}$ and $R_{S_{2}}$ for the scheme B can be calculated as

(12) $$\begin{array}{ccc} R_{S_{1}}^{B} & = & r\left(\frac{M_{2}-1}{N-1},K_{1}K_{2}\right), \end{array}$$

(13) $$\begin{array}{ccc} R_{S_{2}}^{B} & = & r\left(\frac{M_{2}-1}{N-1},K_{2}\right). \end{array}$$

Let us normalize the total file size, mirror memory size, and user cache size involved by scheme A as shown in (14) and (15), respectively,

$$\begin{array}{ccc} F^{\prime } & ≜ & \alpha F, \end{array}$$

(14) $$\begin{array}{ccc} M_{1}^{\prime } & ≜ & \frac{M_{1}F}{F^{\prime }}=\frac{M_{1}}{\alpha }, \end{array}$$

(15) $$\begin{array}{ccc} M_{2}^{\prime } & ≜ & \frac{\beta M_{2}F}{F^{\prime }}=\frac{\beta M_{2}}{\alpha }. \end{array}$$

Moreover, let us normalize user cache size involved by scheme B as shown in (16),

$$\begin{array}{ccc} F^{\prime \prime } & ≜ & (1-\alpha )F, \end{array}$$

(16) $$\begin{array}{ccc} M_{2}^{\prime \prime } & ≜ & \frac{\left(1-\beta \right)M_{2}F}{F^{\prime \prime }}=\frac{\left(1-\beta \right)M_{2}}{1-\alpha }. \end{array}$$

Thus, the secure rates induced by scheme A and scheme B can be normalized with respect to the file size as given by

(17a) $$\begin{array}{ccc} R_{S_{1}}^{A^{\prime }} & = & \alpha K_{2}\cdot r\left(\frac{M_{1}^{\prime }-K_{2}}{\left(N-K_{2}\right)},K_{1}\right)=\alpha K_{2}\cdot r\left(\frac{M_{1}-\alpha K_{2}}{\alpha (N-K_{2})},K_{1}\right), \end{array}$$

(17b) $$\begin{array}{ccc} R_{S_{2}}^{A^{\prime }} & = & \alpha \cdot r\left(\frac{M_{2}^{\prime }-1}{\left(N-1\right)},K_{2}\right)=\alpha \cdot r\left(\frac{\beta M_{2}-\alpha }{\alpha (N-1)},K_{2}\right), \end{array}$$

and

(18a) $$\begin{array}{ccc} R_{S_{1}}^{B^{\prime }} & = & \left(1-\alpha \right)\cdot r\left(\frac{M_{2}^{\prime \prime }-1}{N-1},K_{1}K_{2}\right)=\left(1-\alpha \right)\cdot r\left(\frac{\left(1-\beta \right)M_{2}-\left(1-\alpha \right)}{\left(1-\alpha )(N-1\right)},K_{1}K_{2}\right), \end{array}$$

(18b) $$\begin{array}{ccc} R_{S_{2}}^{B^{\prime }} & = & \left(1-\alpha \right)\cdot r\left(\frac{M_{2}^{\prime \prime }-1}{N-1},K_{2}\right)=\left(1-\alpha \right)\cdot r\left(\frac{\left(1-\beta \right)M_{2}-\left(1-\alpha \right)}{\left(1-\alpha )(N-1\right)},K_{2}\right). \end{array}$$

In the next step, we will calculate $\alpha^{*}$ and $\beta^{*}$ for each of the regimes in a way that both $R_{S_{1}}\left(\alpha ,\beta \right)$ and $R_{S_{2}}\left(\alpha ,\beta \right)$ can be minimized. Let us begin with regime 1. According to (17b) and (18b), for α=β it holds that $R_{S_{2}}\left(\alpha ,\alpha \right)=r\left(\left(M_{2}-1\right)/\left(N-1\right),K_{2}\right)$. It can be verified that α=M/N results in a near-optimal value for $R_{S_{1}}\left(\alpha ,\alpha \right)$ in Regime A. Thus, we chose $\alpha^{*}=M_{1}/N$ and $\beta^{*}=M_{1}/N$ in this regime. Choosing $\alpha^{*}=M_{1}/N$ allows each mirror to store the first part of each of the *N* files in the first transmission. Thus, there will be no need for further transmission between the server and the mirrors in the placement phase or key memory space in the mirrors.

Now let us proceed with regime 2. In this regime, it can be verified from Equation (20b) that $M_{2}<N/K_{2}$ which means that the $M_{2}$ cache area is very small. Thus, $R_{S_{2}}\left(\alpha ,\beta \right)$ will be of order $K_{2}$ for any choice of α and β. Therefore, we only need to choose α and β in a way that $R_{S_{1}}\left(\alpha ,\beta \right)$ is minimized. In this regime, the optimized values for α and β can be obtained as $\alpha^{*}=M_{1}/\left(M_{1}+M_{2}K_{2}\right)$ and $\beta^{*}=M_{1}/\left(M_{2}\left(M_{1}+M_{2}K_{2}\right)\right)$.

In regime 3 (like in regime 1), a choice of $\alpha =\beta =M_{1}/N$ is preferable. However, it should be considered that a large value of β leads to an unacceptably-large value of $R_{S_{1}}\left(\alpha ,\beta \right)$. Thus, a minimum threshold of $\beta^{*}=M_{1}/M_{2}N$ should be considered. Similar to the case of regime 1, no extra transmission between the server and the mirrors in the placement phase or key area in the cache is required in this regime.

After deciding the proper choice of $\alpha^{*},\beta^{*}$, let us calculate $R_{S_{1}}\left(\alpha^{*},\beta^{*}\right)$ and $R_{S_{2}}\left(\alpha^{*},\beta^{*}\right)$ for the generalized scheme as a combination of the secure rates in the two schemes A and B.

**Theorem** **1.** We have the following conditions on $R_{S_{1}}\left(\alpha^{*},\beta^{*}\right)$ and $R_{S_{2}}\left(\alpha^{*},\beta^{*}\right)$

(19a) $$\begin{array}{c} R_{S_{1}}\left(\alpha^{*},\beta^{*}\right)\approx \left\{\begin{array}{cc} \min\left\{K_{1}K_{2},\frac{N-1}{M_{2}-1}\right\} & \mathrm{in}\;\mathrm{regime}\;1,\\ \min\{K_{1}K_{2},\frac{M_{1}}{M_{1}+M_{2}K_{2}}\cdot \frac{K_{2}\left(N-M_{1}\right)}{M_{1}+\left(M_{2}-1\right)K_{2}}\;\\ \;+\frac{M_{2}K_{2}}{M_{1}+M_{2}K_{2}}\;\cdot \frac{\left(N-1\right)K_{2}M_{2}}{\left(M_{2}-1\right)\left(M_{1}+M_{2}K_{2}\right)}\} & \mathrm{in}\;\mathrm{regime}\;2,\\ \frac{{\left(N-M_{1}\right)}^{2}}{N(M_{2}-1)} & \mathrm{in}\;\mathrm{regime}\;3, \end{array}\right. \end{array}$$

*and*

(19b) $$\begin{array}{c} R_{S_{2}}\left(\alpha^{*},\beta^{*}\right)\leq K_{1}\cdot \min\left\{K_{2},\frac{N-1}{M_{2}-1}\right\}. \end{array}$$

**Proof.**  The normalized achievable secure rates for the generalized scheme can be calculated in the form of functions of α and β,

(20a) $$\begin{array}{ccc} R_{S_{1}}\left(\alpha ,\beta \right) & ≜ & R_{S_{1}}^{A^{\prime }}+R_{S_{1}}^{B^{\prime }},\\ & = & \alpha K_{2}\cdot r\left(\frac{M_{1}-\alpha K_{2}}{\alpha (N-K_{2})},K_{1}\right)+\left(1-\alpha \right)\cdot r\left(\frac{\left(1-\beta \right)M_{2}-\left(1-\alpha \right)}{\left(1-\alpha )(N-1\right)},K_{1}K_{2}\right), \end{array}$$

and

(20b) $$\begin{array}{ccc} R_{S_{2}}\left(\alpha ,\beta \right) & ≜ & R_{S_{2}}^{A^{\prime }}+R_{S_{2}}^{B^{\prime }},\\ & = & \alpha \cdot r\left(\frac{\beta M_{2}-\alpha }{\alpha (N-1)},K_{2}\right)+\left(1-\alpha \right)\cdot r\left(\frac{\left(1-\beta \right)M_{2}-\left(1-\alpha \right)}{\left(1-\alpha )(N-1\right)},K_{2}\right). \end{array}$$

With the proper choice of $\alpha^{*},\beta^{*}$, we proceed to calculate secure achievable rates $R_{S_{1}}\left(\alpha^{*},\beta^{*}\right),$$R_{S_{2}}\left(\alpha^{*},\beta^{*}\right)$. As we observe, the secure achievable rates for the generalized caching scheme is a function of r(.,.), as mentioned in the Equation (1). We observe the following,

(21) $$r\left(\frac{M}{N},K\right)\leq \left\{\begin{array}{cc} \min\left\{K,\frac{N}{M}-1\right\} & M\leq N,\;\\ 0 & \mathrm{otherwise}. \end{array}\right.$$

Now let us proceed with calculating the secure achievable rates for each of the regimes of $M_{1}$ and $M_{2}$ beginning with regime 1. According to (5), (20), and (21), the secure achievable rates in this regime can be upper bounded as shown in inequalities (22a) and (22b),

(22a) $$\begin{array}{ccc} R_{S_{1}}\left(\alpha^{*},\beta^{*}\right) & = & \frac{M_{1}}{N}K_{2}\cdot r\left(1,K_{1}\right)+\left(1-\frac{M_{1}}{N}\right)\cdot r\left(\frac{M_{2}-1}{N-1},K_{1}K_{2}\right)\\ & \leq & 0+\min\left\{K_{1}K_{2}\frac{N-1}{M_{2}-1}\right\}\\ & = & \min\left\{K_{1}K_{2},\frac{N-1}{M_{2}-1}\right\}, \end{array}$$

and

(22b) $$\begin{array}{ccc} R_{S_{2}}\left(\alpha^{*},\beta^{*}\right) & = & \frac{M_{1}}{N}\cdot \left(\frac{M_{2}-1}{N-1}\right)+\left(1-\frac{M_{1}}{N}\right)\cdot r\left(\frac{M_{2}-1}{N-1},K_{2}\right)\\ & = & r\left(\frac{M_{2}-1}{N-1}\right)\\ & \leq & \min\left\{K_{2},\frac{N-1}{M_{2}-1}\right\}. \end{array}$$

Through a similar reasoning, the upper bounds to the secure achievable rate $R_{S_{1}}\left(\alpha^{*},\beta^{*}\right)$ in regime 2 can be obtained from the inequality (23a) (the form of equations are different from regime 1).

(23a) $$\begin{array}{ccc} R_{S_{1}}\left(\alpha^{*},\beta^{*}\right) & = & \frac{M_{1}K_{2}}{M_{1}+M_{2}K_{2}}\cdot r\left(\frac{M_{1}+M_{2}K_{2}-K_{2}}{N-K_{2}},K_{1}\right)\\ & & +\frac{M_{2}K_{2}}{M_{1}+M_{2}K_{2}}\cdot r\left(\frac{\left(M_{2}-1\right)\left(M_{1}+M_{2}K_{2}\right)}{\left(N-1\right)M_{2}K_{2}},K_{1}K_{2}\right)\\ & \leq & \frac{M_{1}K_{2}}{M_{1}+K_{2}M_{2}}\cdot \min\left\{K_{1},\frac{N-K_{2}}{M_{1}+M_{2}K_{2}-K_{2}}-1\right\}\\ & & +\frac{M_{2}K_{2}}{M_{1}+M_{2}K_{2}}\cdot \min\left\{K_{1}K_{2},\frac{\left(N-1\right)K_{2}M_{2}}{\left(M_{2}-1\right)\left(M_{1}+M_{2}K_{2}\right)}-1\right\}\\ & \leq & \frac{M_{1}}{M_{1}+M_{2}K_{2}}\cdot \min\left\{K_{1}K_{2},\frac{K_{2}\left(N-M_{1}\right)}{M_{1}+\left(M_{2}-1\right)K_{2}}\right\}\\ & & +\frac{M_{2}K_{2}}{M_{1}+M_{2}K_{2}}\cdot \min\left\{K_{1}K_{2},\frac{\left(N-1\right)K_{2}M_{2}}{\left(M_{2}-1\right)\left(M_{1}+M_{2}K_{2}\right)}\right\}\\ & \leq & \min\left\{K_{1}K_{2},\frac{M_{1}}{M_{1}+M_{2}K_{2}}\cdot \frac{K_{2}\left(N-M_{1}\right)}{M_{1}+\left(M_{2}-1\right)K_{2}}\right.\\ & & \left.+\frac{M_{2}K_{2}}{M_{1}+M_{2}K_{2}}\cdot \frac{\left(N-1\right)K_{2}M_{2}}{\left(M_{2}-1\right)\left(M_{1}+M_{2}K_{2}\right)}\right\}. \end{array}$$

Furthermore, according to (20) and (21), the reader can easily verify that $R_{S_{2}}\left(\alpha^{*},\beta^{*}\right)$ in regime 2 will be upper bounded by

(23b) $$\begin{array}{c} R_{S_{2}}\left(\alpha^{*},\beta^{*}\right)\leq K_{2}=\min\left\{K_{2},\frac{N-1}{M_{2}-1}\right\}. \end{array}$$

Additionally, for regime 3 we have,

(24a) $$\begin{array}{ccc} R_{S_{1}}\left(\alpha^{*},\beta^{*}\right) & = & \left(1-\frac{M_{1}}{N}\right)\cdot r\left(1-\frac{M_{1}}{N}\right)+r\left(\frac{\left(1-1/K_{1}\right)M_{2}-\left(1-M_{1}/N\right)}{\left(1-M_{1}/N\right)\left(N-1\right)},K_{2}\right)\\ & \leq & 0+\left(1-\frac{M_{1}}{N}\right)\cdot \min\left\{K_{1}K_{2},\frac{\left(N-M_{1}\right)\left(N-1\right)K_{1}}{NM_{2}\left(K_{1}-1\right)-K_{1}\left(N-M_{1}\right)}\right\}\\ & \leq & \left(\frac{N-M_{1}}{N}\right)\cdot \frac{K_{1}\left(N-M_{1}\right)\left(N-1\right)}{\left(K_{1}-1\right)N\left(M_{2}-1\right)}\\ & \leq & \frac{K_{1}}{K_{1}-1}\cdot \frac{{\left(N-M_{1}\right)}^{2}}{N(M_{2}-1)}, \end{array}$$

and

(24b) $$\begin{array}{ccc} R_{S_{2}}\left(\alpha^{*},\beta^{*}\right) & = & \frac{M_{1}}{N}\cdot r\left(\frac{M_{2}}{K_{1}M_{1}},K_{2}\right)+\left(1-\frac{M_{1}}{N}\right)\cdot r\left(\frac{\left(1-1/K_{1}\right)M_{2}-\left(1-M_{1}/N\right)}{\left(1-M_{1}/N\right)\left(N-1\right)},K_{2}\right)\\ & \leq & \frac{M_{1}}{N}\cdot \min\left\{K_{2},\frac{K_{1}\left(N-1\right)}{M_{2}-1}\right\}+\left(1-\frac{M_{1}}{N}\right)\min\left\{K_{2},\frac{K_{1}\left(N-1\right)}{M_{2}-1}\right\}\\ & \leq & \min\left\{K_{2},\frac{K_{1}\left(N-1\right)}{\left(M_{2}-1\right)}\right\}\\ & \leq & K_{1}\cdot \min\left\{K_{2},\frac{N-1}{M_{2}-1}\right\}. \end{array}$$

Summarizing our results for the generalized scheme from the discussions above we have

(25a) $$\begin{array}{c} R_{S_{1}}\left(\alpha^{*},\beta^{*}\right)\approx \left\{\begin{array}{cc} \min\left\{K_{1}K_{2},\frac{N-1}{M_{2}-1}\right\} & \mathrm{in}\;\mathrm{regime}\;1,\\ \min\{K_{1}K_{2},\frac{M_{1}}{M_{1}+M_{2}K_{2}}\cdot \frac{K_{2}\left(N-M_{1}\right)}{M_{1}+\left(M_{2}-1\right)K_{2}}\;\\ \;+\frac{M_{2}K_{2}}{M_{1}+M_{2}K_{2}}\;\cdot \frac{\left(N-1\right)K_{2}M_{2}}{\left(M_{2}-1\right)\left(M_{1}+M_{2}K_{2}\right)}\} & \mathrm{in}\;\mathrm{regime}\;2,\\ \frac{{\left(N-M_{1}\right)}^{2}}{N(M_{2}-1)} & \mathrm{in}\;\mathrm{regime}\;3, \end{array}\right. \end{array}$$

and

(25b) $$\begin{array}{c} R_{S_{2}}\left(\alpha^{*},\beta^{*}\right)\leq K_{1}\cdot \min\left\{K_{2},\frac{N-1}{M_{2}-1}\right\}. \end{array}$$

The proof is now complete. □

### 4.3. Memory Requirements

An information-theoretical analysis will reveal some minimum requirements regarding the cache size in the mirrors and uses cache. Consider a caching system in which two files denoted by A and B are residing in the main server N=2. Consider $K_{1}=2$ mirrors denoted by $m_{1}$ and $m_{2}$ with each of mirrors cache size $M_{1}$. Cache contents denoted by $Z_{1}^{m}$, $Z_{2}^{m}$ cached by users in the placement phase. Let us assume that mirror $m_{1}$ demands a content item $A_{\alpha }$ in the delivery phase which is part of file A and mirror $m_{2}$ demands part of file B denoted by $B_{\alpha }$. Both demanded items are assumed to be of size αF which can be considered a fraction equal to α of the size of file A or B. The mentioned demands can be represented by a demand vector $\left(d_{1},d_{2}\right)=\left(A_{\alpha },B_{\alpha }\right)$.

In this setup, the main server will transmit $X_{\left(A_{\alpha },B_{\alpha }\right)}$ to the mirrors which should be capable of regenerating the items $A_{\alpha }$ and $B_{\alpha }$ when combined with $Z_{1}^{m}$ and $Z_{2}^{m}$. From an information theoretical point of view, the criterion stated by inequality (26) should hold to make it possible to achieve this goal,

(26) $$H(A_{\alpha },B_{\alpha }|X_{\left(A_{\alpha },B_{\alpha }\right)},Z_{1}^{m},Z_{2}^{m})\leq \epsilon .$$

For the security constraint between the server and the mirrors, inequality (27) should hold in order to keep the delivery phase transmissions confidential,

(27) $$I(A_{\alpha },B_{\alpha };X_{\left(A_{\alpha },B_{\alpha }\right)})\leq \delta .$$

Using (26) and (27) we have

(28) $$\begin{array}{ccc} 2\alpha F & \leq & H(A_{\alpha },B_{\alpha }),\\ & = & I\left(A_{\alpha },B_{\alpha };X_{\left(A_{\alpha },B_{\alpha }\right)},Z_{1}^{m},Z_{2}^{m}\right)+H\left(A_{\alpha },B_{\alpha }|X_{\left(A_{\alpha },B_{\alpha }\right)},Z_{1}^{m},Z_{2}^{m}\right),\\ & \leq & I(A_{\alpha },B_{\alpha };X_{\left(A_{\alpha },B_{\alpha }\right)},Z_{1}^{m},Z_{2}^{m})+\epsilon ,\\ & = & I(A_{\alpha },B_{\alpha };X_{\left(A_{\alpha },B_{\alpha }\right)}+I\left(A_{\alpha },B_{\alpha };Z_{1}^{m},Z_{2}^{m}|X_{\left(A_{\alpha },B_{\alpha }\right)}\right)+\epsilon ,\\ & \leq & I(A_{\alpha },B_{\alpha };Z_{1}^{m},Z_{2}^{m}|X_{\left(A_{\alpha },B_{\alpha }\right)})+\delta +\epsilon ,\\ & \leq & H(Z_{1}^{m},Z_{2}^{m}|X_{\left(A_{\alpha },B_{\alpha }\right)})+\delta +\epsilon ,\\ & \leq & 2M_{1}F+\delta +\epsilon . \end{array}$$

From (28) we immediately obtain

(29) $$M_{1}\geq \alpha -\frac{\delta }{F}-\frac{\epsilon }{F}.$$

When δ and ϵ approach zero, inequality (29) will be converted to $M_{1}\geq \alpha$. Again, we note that users should be able to recover both file A and file B from a single cached item $Z_{1}^{m}$ along with two items within $X_{\left(A_{\alpha },B_{\alpha }\right)}$ and $X_{\left(B_{\alpha },A_{\alpha }\right)}$ transmitted in response to the demand vectors $\left(d_{1},d_{2}\right)=\left(A_{\alpha },B_{\alpha }\right)$ and $\left(d_{1},d_{2}\right)=\left(B_{\alpha },A_{\alpha }\right)$, respectively. The latter requirement leads to the following inequalities,

(30) $$\begin{array}{cc} \; & H(A_{\alpha },B_{\alpha }|X_{\left(A_{\alpha },B_{\alpha }\right)},X_{\left(B_{\alpha },A_{\alpha }\right)},Z_{1}^{m})\leq \epsilon , \end{array}$$

(31) $$\begin{array}{cc} \; & I(A_{\alpha },B_{\alpha };X_{\left(A_{\alpha },B_{\alpha }\right)})\leq \delta . \end{array}$$

Through similar reasoning the latter two inequalities will lead to $R_{s}^{*}+M_{1}\geq 2\alpha$ where $R_{s}^{*}$ is minimum rate between the server and the mirrors.

### 4.4. Lower Bounds

Now let us discuss the lower bounds on secure rates $R_{S_{1}}$ and $R_{S_{2}}$ for different values of $M_{1}$, $M_{2}$ given the feasibility of $\left(M_{1},M_{2},R_{S_{1}},R_{S_{2}}\right)$. To do this, we follow the approach taken in [3] for the secure non-hierarchical scheme and extend the discussions and results to the case of the secure hierarchical network.

**Theorem** **2.** *We have*

$$\begin{array}{ccc} R_{S_{1}} & \geq & \max_{\begin{array}{c} s_{1}\in \left\{1,2,\ldots ,K_{1}\right\}\\ s_{2}\in \left\{1,2,\ldots ,K_{2}\right\} \end{array}}max\left\{s_{1}s_{2}\left[1-\frac{1}{N-2s_{1}s_{2}}\left(s_{1}M_{1}+s_{1}s_{2}\left(M_{2}-1\right)\right)\right],\frac{s_{1}s_{2}\left(N-s_{1}M_{1}-s_{1}s_{2}M_{2}\right)}{N}\right\}\\ & ≜ & R_{S_{1}}^{lb}\left(M_{1},M_{2}\right), \end{array}$$

*and*

$$\begin{array}{cc} & R_{S_{2}}\;\;\geq \;\;\max_{t\in \{1,2,\ldots ,K_{2}\}}\frac{t(N-tM_{2})}{N}. \end{array}$$

**Proof.**  Let us begin with the lower bound on $R_{S_{1}}$. For $s_{1}\in \left\{1,2,\ldots ,K_{1}\right\}$ and $s_{2}\in \left\{1,2,\ldots ,K_{2}\right\}$, suppose the first $s_{1}$ mirrors store $Z_{1}^{m},Z_{2}^{m},\cdots ,Z_{s_{1}}^{m}$. Furthermore, assume that for $i\in \{1,2,\ldots ,s_{1}\}$ and $j\in \{1,2,\ldots ,s_{2}\}$, every user $C_{\left(i,j\right)}$ caches $Z_{i,1}^{u},Z_{i,2}^{u},\cdots ,Z_{i,s_{2}}^{u}$. Suppose the mentioned users issue the demand matrix $D^{1}$ defined as $d_{i,j}^{1}=\left(i-1\right)s_{2}+j$ which includes requests for the first $s_{1}s_{2}$ files residing in the main server. The items transmitted by the main server within $X_{1}=X_{\left(d_{1,1},\ldots ,d_{s_{1}s_{2}}\right)}$, along with the mirrored items $Z_{1}^{m},Z_{2}^{m},\ldots ,Z_{s_{1}}^{m}$ and cached items $Z_{i,1}^{u},Z_{i,2}^{u},\ldots ,Z_{i,s_{2}}^{u}$ must able to decode the files $W_{1},W_{2},\ldots ,W_{s_{1}s_{2}}$.

Similarly, for the different request matrix D, where user $U_{\left(i,j\right)}$ demands $d_{i,j}=s_{1}s_{2}+\left(i-1\right)s_{2}+j$, i.e., requesting next $s_{1}s_{2}$ files from the server. The transmission $X_{2}$, along with mirrors $Z_{i,1}^{m},Z_{i,2}^{m},\ldots ,Z_{i,s_{2}}^{m}$ and users cache $Z_{i,1}^{u},Z_{i,2}^{u},\ldots ,Z_{i,s_{2}}^{u}$ must be able to decode the files $W_{s_{1}s_{2}+1}$, $W_{s_{1}s_{2}+2},$ …, $W_{2s_{1}s_{2}}$. Likewise, considering all $\left\lfloor N/s_{1}s_{2}\right\rfloor$ request matrices, multicast transmission $X_{1},\ldots ,X_{\left\lfloor N/s_{1}s_{2}\right\rfloor }$ along with mirrors $Z_{1}^{m},Z_{2}^{m},\ldots \;Z_{s_{1}}^{m}$ and users cache $Z_{i,1}^{u},Z_{i,2}^{u},\ldots ,Z_{i,s_{2}}^{u}$, must be able to recover the files $W_{1},\ldots ,W_{s_{1}s_{2}\left\lfloor N/s_{1}s_{2}\right\rfloor }$. Let

$$\begin{array}{ccc} \tilde{W} & = & \left\{W_{1},\ldots ,W_{s_{1}s_{2}\left\lfloor N/s_{1}s_{2}\right\rfloor }\right\}\\ \tilde{X} & = & \left\{X_{1},\ldots ,X_{\left\lfloor N/s_{1}s_{2}\right\rfloor }\right\}\\ \tilde{X}\setminus \{l\} & = & \left\{X_{1},\ldots ,X_{l-1},X_{l+1},\ldots ,X_{\left\lfloor N/s_{1}s_{2}\right\rfloor }\right\}\\ {\tilde{Z}}^{m} & = & \left\{Z_{1}^{m},\ldots ,Z_{s_{1}}^{m}\right\}={\tilde{Z}}_{i}^{m}\\ {\tilde{Z}}^{u} & = & \left\{Z_{1,1}^{u},\ldots ,Z_{1,s_{2}}^{u},Z_{2,1}^{u},\ldots ,Z_{s_{1}s_{2}}^{u}\right\}=\left\{Z_{i,j}^{u}\right\}. \end{array}$$

Another point implied by the feasibility of $\left(M_{1},M_{2},R_{s_{1}},R_{s_{2}}\right)$ in our system model is that the external adversary should not be able to retrieve any information regarding the contents being transmitted in the delivery phase. This criterion is formally described by inequalities (32) and (33),

(32) $$\begin{array}{c} H\left(\tilde{W}|\tilde{X},{\tilde{Z}}^{m},{\tilde{Z}}^{u}\right)\leq \epsilon_{1}, \end{array}$$

and

(33) $$\begin{array}{c} I\left(\tilde{W};X_{l}\right)\leq \epsilon_{2},\;l=1,\ldots ,q. \end{array}$$

Consider the information flow consisting of multicast transmission $X_{1},\ldots ,X_{\left\lfloor N/s_{1}s_{2}\right\rfloor }$, mirrors $Z_{1},Z_{2},\ldots ,Z_{s_{1}}$ and users cache $Z_{i,1}\ldots ,Z_{i,s_{2}}$ for decoding file $W_{1},\ldots ,W_{s_{1}s_{2}\left\lfloor N/s_{1}s_{2}\right\rfloor }$. We have

(34) $$\begin{array}{ccc} s_{1}s_{2}\left\lfloor N/s_{1}s_{2}\right\rfloor F & \leq & H(\tilde{W})\\ & = & I\left(\tilde{W};\tilde{X},{\tilde{Z}}^{u},{\tilde{Z}}^{m}\right)+H\left(\tilde{W}|\tilde{X},{\tilde{Z}}^{u},{\tilde{Z}}^{m}\right)\\ & \leq & I\left(\tilde{W};\tilde{X},{\tilde{Z}}^{u},{\tilde{Z}}^{m}\right)+\epsilon_{1}\\ & = & I\left(\tilde{W};X_{l}\right)+I\left(\tilde{W};\tilde{X}\setminus \{l\},{\tilde{Z}}^{m},{\tilde{Z}}^{u}|X_{l}\right)+\epsilon_{1}\\ & \leq & I\left(\tilde{W};\tilde{X}\setminus \{l\},{\tilde{Z}}^{m},{\tilde{Z}}^{u}|X_{l}\right)+\epsilon_{1}+\epsilon_{2}\\ & \leq & H(\tilde{X}\setminus \{l\},{\tilde{Z}}^{m},Z^{u})+\epsilon \\ & \leq & \sum_{k=1,k\neq l}^{\left\lfloor N/s_{1}s_{2}\right\rfloor }H\left(X_{k}\right)+\sum_{i=1}^{s_{1}}H\left(Z_{i}^{m}\right)+\sum_{i=1}^{s_{1}}\sum_{j=1}^{s_{2}}H\left(Z_{i,j}^{u}\right)+\epsilon \\ & \leq & \left(\left\lfloor N/s_{1}s_{2}\right\rfloor -1\right)R_{s_{1}}F+s_{1}M_{1}F+s_{1}s_{2}M_{2}F+\epsilon . \end{array}$$

So,

(35) $$\begin{array}{c} s_{1}s_{2}\left\lfloor N/s_{1}s_{2}\right\rfloor \leq \left(\left\lfloor N/s_{1}s_{2}\right\rfloor -1\right)R_{S_{1}}+s_{1}M_{1}+s_{1}s_{2}M_{2}+\frac{\epsilon }{F}. \end{array}$$

Solving and optimizing for all possible values of $s_{1}$ and $s_{2}$ we obtain

(36) $$\begin{array}{ccc} R_{S_{1}} & \geq & \max_{\begin{array}{c} s_{1}\in \left\{1,2,\ldots ,K_{1}\right\}\\ s_{2}\in \left\{1,2,\ldots ,K_{2}\right\} \end{array}}\lim_{\epsilon \to 0}\frac{1}{\lfloor N/s_{1}s_{2}\rfloor -1}\left\{s_{1}s_{2}\left\lfloor N/s_{1}s_{2}\right\rfloor -s_{1}M_{1}-s_{1}s_{2}M_{2}-\frac{\epsilon }{F}\right\}\\ & \geq & \max_{\begin{array}{c} s_{1}\in \left\{1,2,\ldots ,K_{1}\right\}\\ s_{2}\in \left\{1,2,\ldots ,K_{2}\right\} \end{array}}s_{1}s_{2}-\frac{s_{1}M_{1}+s_{1}s_{2}\left(M_{2}-1\right)}{N/s_{1}s_{2}-2}\\ & \geq & \max_{\begin{array}{c} s_{1}\in \left\{1,2,\ldots ,K_{1}\right\}\\ s_{2}\in \left\{1,2,\ldots ,K_{2}\right\} \end{array}}s_{1}s_{2}\left(1-\frac{s_{1}M_{1}+s_{1}s_{2}\left(M_{2}-1\right)}{N-2s_{1}s_{2}}\right). \end{array}$$

We can obtain an alternate lower bound by using $\left\lceil N/s_{1}s_{2}\right\rceil$ transmissions instead of $\left\lfloor N/s_{1}s_{2}\right\rfloor$ in (35),

(37) $$\begin{array}{ccc} R_{S_{1}} & \geq & \max_{\begin{array}{c} s_{1}\in \left\{1,2,\ldots ,K_{1}\right\}\\ s_{2}\in \left\{1,2,\ldots ,K_{2}\right\} \end{array}}\frac{1}{\lceil N/s_{1}s_{2}\rceil -1}\left(N-s_{1}M_{1}-s_{1}s_{2}M_{2}\right)\\ & \geq & \max_{\begin{array}{c} s_{1}\in \left\{1,2,\ldots ,K_{1}\right\}\\ s_{2}\in \left\{1,2,\ldots ,K_{2}\right\} \end{array}}\frac{1}{N/s_{1}s_{2}}\left(N-s_{1}M_{1}-s_{1}s_{2}M_{2}\right)\\ & \geq & \max_{\begin{array}{c} s_{1}\in \left\{1,2,\ldots ,K_{1}\right\}\\ s_{2}\in \left\{1,2,\ldots ,K_{2}\right\} \end{array}}\frac{1}{N}\left(s_{1}s_{2}\left(N-s_{1}M_{1}-s_{1}s_{2}M_{2}\right)\right). \end{array}$$

The inequalities (36) and (37) hold for any value of $s_{1}\in \left\{1,2,\ldots ,K_{1}\right\}$ and $s_{2}\in \left\{1,2,\ldots ,K_{2}\right\}$. So, we obtain the following lower bound on $R_{S_{1}}$ for the tuple $\left(M_{1},M_{2},R_{S_{1}},R_{S_{2}}\right)$ to be feasible,

(38) $$\begin{array}{ccc} R_{S_{1}} & \geq & \max_{\begin{array}{c} s_{1}\in \left\{1,2,\ldots ,K_{1}\right\}\\ s_{2}\in \left\{1,2,\ldots ,K_{2}\right\} \end{array}}max\left\{s_{1}s_{2}\left[1-\frac{1}{N-2s_{1}s_{2}}\left(s_{1}M_{1}+s_{1}s_{2}\left(M_{2}-1\right)\right)\right],\frac{s_{1}s_{2}\left(N-s_{1}M_{1}-s_{1}s_{2}M_{2}\right)}{N}\right\}\\ & ≜ & R_{S_{1}}^{lb}\left(M_{1},M_{2}\right). \end{array}$$

After calculating the lower bound for $R_{S_{1}}$, let us proceed with that of $R_{S_{2}}$ assuming the feasibility of $\left(M_{1},M_{2},R_{S_{1}},R_{S_{2}}\right)$. Let $t\in \{1,2,\ldots ,K_{2}\}$. Consider the *t* users cache $U_{\left(1,j\right)}$ as $Z_{1,1}^{u},Z_{1,2}^{u},\ldots ,Z_{1,t}^{u}$ with j∈{1,2,…,t}. Consider the request matrix D with demands $d_{1,j}=j$, i.e., requesting *t* files from the server. The transmission $Y_{1}=Y_{\left(d_{1,1},\ldots ,d_{1,t}\right)}$, along with the users cache $U_{\left(1,j\right)}$$Z_{1,1}^{u},Z_{1,2}^{u},\ldots ,Z_{1,t}^{u}$ must be able to decode the files $W_{1},\ldots ,W_{t}$. Similarly, for the different request matrix D, where the user demands $d_{i,j}=t+j$, i.e., requesting another *t* files from the server. The transmission $Y_{2}$ along with the users cache $U_{1,j}$, must be able to decode the files $W_{t+1},\ldots ,W_{2t}$. Likewise, considering the all ⌈N/t⌉ request matrices, multicast transmission, $Y_{1},\ldots ,Y_{\left\lceil N/t\right\rceil }$ along with the users cache $Z_{1,1}^{u},Z_{1,2}^{u},\ldots ,Z_{1,t}^{u}$ must be able to recover the files $W_{1},\ldots ,W_{N}$. Assuming $Y_{l}$ be the information leaked to the external adversary through the link connecting between the mirror and its corresponding users.

$$\begin{array}{ccc} \tilde{W} & = & \left\{W_{1},\ldots ,W_{N}\right\}\\ \tilde{Y} & = & \left\{Y_{1},\ldots ,Y_{\left\lceil N/t\right\rceil }\right\}\\ \tilde{Y}\setminus \{l\} & = & \left\{Y_{1},\ldots ,Y_{l-1},Y_{l+1},\ldots ,Y_{\left\lceil N/t\right\rceil }\right\}\\ {\tilde{Z}}^{u} & = & \left\{Z_{1,1}^{u},\ldots ,Z_{1,t}^{u}\right\}=\left\{Z_{1,j}^{u}\right\},\;where\;j\in \left\{1,2,\ldots ,t\right\}. \end{array}$$

The file recovery and security constraints can be stated as

(39) $$\begin{array}{ccc} H(\tilde{W}|\tilde{Y},{\tilde{Z}}^{u}) & \leq & \epsilon_{1}, \end{array}$$

(40) $$\begin{array}{ccc} I(\tilde{W};Y_{l}) & \leq & \epsilon_{2},\;l=1,\ldots ,Y_{\left\lceil N/t\right\rceil } \end{array}$$

This is similar case of single layer secure scheme. Consider the information flow consisting of multicast transmission $Y_{1},\ldots ,Y_{\left\lceil N/t\right\rceil }$ and users cache $Z_{1,1}^{u},Z_{1,2}^{u},\ldots ,Z_{1,t}^{u}$ for decoding the files $W_{1},W_{2},\ldots ,W_{N}$. We have

(41) $$\begin{array}{ccc} NF & = & H(\tilde{W})\\ & = & I\left(\tilde{W};\tilde{Y},{\tilde{Z}}^{u}\right)+\left(\tilde{W}|\tilde{Y},{\tilde{Z}}^{u}\right)\\ & \leq & I\left(\tilde{W};\tilde{Y},{\tilde{Z}}^{u}\right)+\epsilon_{1}\\ & = & I\left(\tilde{W};Y_{l}\right)+I\left(\tilde{W};\tilde{Y}\setminus \{l\},{\tilde{Z}}^{u}|Y_{l}\right)+\epsilon_{1}\\ & \leq & I\left(\tilde{W};\tilde{Y}\setminus \{l\},{\tilde{Z}}^{u}|Y_{l}\right)+\epsilon_{1}+\epsilon_{2}\\ & \leq & H\left(\tilde{Y}\setminus \{l\},{\tilde{Z}}^{u}\right)+\epsilon ,\;where\;\epsilon_{1}+\epsilon_{2}=\epsilon \\ & \leq & \sum_{i=1,i\neq l}^{\left\lceil N/t\right\rceil }H\left(Y_{i}\right)+\sum_{j=1}^{t}H\left(Z_{1,j}^{u}\right)+\epsilon \\ & \leq & \left(\left\lceil N/t\right\rceil -1\right)R_{S_{2}}F+tM_{2}F+\epsilon . \end{array}$$

Therefore,

(42) $$\begin{array}{c} N=\left(\left\lceil N/t\right\rceil -1\right)R_{S_{2}}+tM_{2}+\frac{\epsilon }{F}. \end{array}$$

Solving and optimizing for all value of *t*, we obtain the following lower bound

(43) $$\begin{array}{ccc} R_{S_{2}} & \geq & \max_{t\in \{1,2,\ldots ,K_{2}\}}\lim_{\epsilon \to 0}\frac{N-tM_{2}-\frac{2\epsilon }{F}}{\left\lceil N/t\right\rceil -1}\\ & \geq & \max_{t\in \{1,2,\ldots ,K_{2}\}}\frac{N-tM_{2}}{N/t}\\ & = & \max_{t\in \{1,2,\ldots ,K_{2}\}}\frac{t(N-tM_{2})}{N}\\ & ≜ & R_{S_{2}}^{lb}\left(M_{1},M_{2}\right). \end{array}$$

□

## 5. Gap Analysis

In this section, we analyze the gap between the secure achievable rates and the corresponding lower bounds.

### 5.1. $R_{S_{1}}\left(\alpha^{*},\beta^{*}\right)$ against $R_{S_{1}}^{lb}\left(M_{1},M_{2}\right)$

**Theorem** **3.** $R_{S_{1}}\left(\alpha^{*},\beta^{*}\right)$ will vary between a constant multiplicative and a constant additive gap with $R_{S_{1}}^{lb}\left(M_{1},M_{2}\right)$. Specifically,

$$R_{S_{1}}\geq R_{S_{1}}^{lb}\left(M_{1},M_{2}\right)\geq \frac{1}{96}R_{S_{1}}\left(\alpha^{*},\beta^{*}\right)-4.$$

**Proof.**  The values of $\alpha^{*}$ and $\beta^{*}$, and consequently $R_{s_{1}}\left(\alpha^{*},\beta^{*}\right)$ obviously depend on the regime characterized by $M_{1}$ and $M_{2}$. This makes it necessary to examine each of the regimes. We begin with regime 1 assuming that $N\geq K_{1}K_{2}$, $K_{1}\geq 4$ and $K_{2}\geq 4$.

Regime 1: $M_{1}+M_{2}K_{2}\geq N$ and $0\leq M_{1}\leq \frac{N}{K_{1}}$ where $M_{1}\geq \frac{M_{1}K_{2}}{N}$, $M_{2}\geq 1$. Inequalities (25a) and (38) give the achievable secure rate $R_{S_{1}}\left(\alpha^{*},\beta^{*}\right)$, as well as the lower bound on $R_{S_{1}}\left(M_{1},M_{2}\right)$ for regime 1.

In order to make the margin of the gap more manageable, we further divide our discussions regarding this regime into three sub-regimes specified as follows:

$$\begin{array}{ccc} 1.A) & & \frac{M_{1}K_{2}}{N}\leq M_{1}\leq \frac{N}{2K_{1}},\;\frac{3N}{4K_{2}}\leq M_{2}\leq \frac{N}{4},\\ 1.B) & & \frac{N}{2K_{1}}\leq M_{1}\leq \frac{N}{K_{1}},\;\frac{3N}{4K_{2}}\leq M_{2}\leq \frac{N}{4},\\ 1.C) & & \frac{M_{1}K_{2}}{N}\leq M_{1}\leq \frac{N}{K_{1}},\;\frac{N}{4}\leq M_{2}\leq N. \end{array}$$

For sub-regime 1.A), let us feed $s_{1}=1$ and $s_{2}=\left\lfloor \frac{N}{2M_{2}}\right\rfloor$ (which is a valid choice because ⌊z⌋≥z/2 for any z≥1) into (38) which gives

(44) $$\begin{array}{ccc} R_{S_{1}}^{lb}\left(M_{1},M_{2}\right) & \geq & \frac{\left\lfloor \frac{N}{2M_{2}}\right\rfloor \left(N-M_{1}-\left\lfloor \frac{N}{2M_{2}}\right\rfloor M_{2}\right)}{N}\\ & \overset{\left(a\right)}{\geq } & \frac{1}{N}\left[\frac{N}{4M_{2}}\left(N-\frac{N}{2K_{1}}-\frac{N}{2M_{2}}\cdot M_{2}\right)\right]\\ & \geq & \frac{N}{4M_{2}}\left(1-\frac{1}{2K_{1}}-\frac{1}{2}\right)\\ & \overset{\left(b\right)}{\geq } & \frac{N}{4M_{2}}\left(\frac{1}{2}-\frac{1}{8}\right)\\ & \overset{\left(c\right)}{\geq } & \frac{3N}{32M_{2}}\geq \frac{3}{32}\cdot \frac{4}{5}\cdot \frac{N-1}{M_{2}-1},\\ & \geq & \frac{3}{40}\min\left\{K_{1}K_{2},\frac{N-1}{M_{2}-1}\right\}. \end{array}$$

In deriving (44), we have used (a):⌊z⌋≥z/2∀z≥1, $\left(b\right):K_{1}\geq 4$ and $\left(c\right):N\geq K_{1}K_{2}$. Combining (44) and (22a), we obtain

(45) $$R_{S_{1}}^{lb}\left(M_{1},M_{2}\right)\geq \frac{3}{40}R_{S_{1}}\left(\alpha^{*},\beta^{*}\right).$$

For sub-regime 1.B, let

$$\left(s_{1},s_{2}\right)=\left\{\begin{array}{cc} \left(\left\lfloor \frac{N}{K_{1}M_{1}}\right\rfloor ,\left\lfloor \frac{M_{1}}{M_{2}}\right\rfloor \right) & \mathrm{for}\;M_{1}\geq M_{2},\;\\ \left(\left\lfloor \frac{N}{K_{1}M_{1}}\right\rfloor ,1\right) & \mathrm{otherwise}. \end{array}\right.$$

Note that for $M_{1}\geq M_{2}$, we have

$$\begin{array}{ccc} 1 & = & \left\lfloor \frac{N}{K_{1}.N/K_{1}}\right\rfloor \leq \left\lfloor \frac{N}{K_{1}M_{1}}\right\rfloor \leq \frac{N}{K_{1}M_{1}}\leq 2,\\ 1 & \leq & \left\lfloor \frac{M_{1}}{M_{2}}\right\rfloor \leq \frac{M_{1}}{M_{2}}\leq \frac{N/K_{1}}{3N/(4K_{2})}=\frac{4K_{2}}{3K_{1}}, \end{array}$$

and for $M_{1}<M_{2}$, we have

$$\begin{array}{c} 1=\left\lfloor \frac{N}{4N/4}\right\rfloor \leq \left\lfloor \frac{N}{4M_{2}}\right\rfloor \leq \left\lfloor \frac{N}{K_{1}M_{1}}\right\rfloor \leq \frac{N}{K_{1}M_{1}}\leq 2. \end{array}$$

Finally, feeding the chosen values of $s_{1},s_{2}$ into (38) we obtain

(46) $$\begin{array}{ccc} R_{S_{1}}^{lb}\left(M_{1},M_{2}\right) & \geq & \frac{\frac{N}{4K_{1}M_{2}}\left(N-\frac{N}{K_{1}M_{1}}\cdot M_{1}-\frac{N}{4M_{2}}\cdot M_{2}\right)}{N}\\ & \geq & \frac{N}{4K_{1}M_{2}}\left(1-\frac{1}{K_{1}}-\frac{1}{4}\right)\\ & \geq & \frac{N}{32M_{2}}\geq \frac{1}{32}\cdot \frac{4}{5}\cdot \frac{N-1}{M_{2}-1}\\ & \geq & \frac{1}{40}\min\left\{K_{1}K_{2},\frac{N-1}{M_{2}-1}\right\}. \end{array}$$

Combining (46) and (22a), we obtain

(47) $$R_{S_{1}}^{lb}\left(M_{1},M_{2}\right)\geq \frac{1}{40}R_{S_{1}}\left(\alpha^{*},\beta^{*}\right).$$

Similarly, in sub-regime 1.C, we have

(48) $$\begin{array}{ccc} R_{S_{1}}^{lb}\left(M_{1},M_{2}\right) & \geq & \frac{N}{M_{2}}-4\;\geq \;\frac{N-1}{M_{2}-1}-4\\ & \geq & \min\left\{K_{1}K_{2},\frac{N-1}{M_{2}-1}\right\}-4. \end{array}$$

Combining (48) and (22a), we obtain

(49) $$R_{S_{1}}^{lb}\left(M_{1},M_{2}\right)\geq R_{S_{1}}\left(\alpha^{*},\beta^{*}\right)-4.$$

Our analysis for sub-regimes 1.A, 1.B and 1.C demonstrate that the secure achievable rate $R_{S_{1}}^{lb}\left(M_{1},M_{2}\right)$ is within a constant multiplicative and additive gap for regime 1.

As for regime 2, we further divide it into the following sub-regimes.

$$\begin{array}{ccc} \left(2.A\right) & & \frac{M_{1}K_{2}}{M_{1}+M_{2}K_{2}}\leq M_{1}<\frac{N}{K_{1}},\;1\leq M_{2}<\frac{N}{K_{1}K_{2}},\\ \left(2.B\right) & & \frac{M_{1}K_{2}}{M_{1}+M_{2}K_{2}}\leq M_{1}<\frac{N}{K_{1}},\;\frac{N}{K_{1}K_{2}}\leq M_{2}<\frac{N}{3K_{2}},\\ \left(2.C\right) & & \frac{M_{1}K_{2}}{M_{1}+M_{2}K_{2}}\leq M_{1}<\frac{N}{K_{1}},\;\frac{N}{3K_{2}}\leq M_{2}<\frac{N}{4},\\ \left(2.D\right) & & \frac{N}{K_{1}}\leq M_{1}\leq N,\;1\leq M_{2}<\frac{N-M_{1}}{2K_{2}},\\ \left(2.E\right) & & \frac{N}{K_{1}}\leq M_{1}\leq N,\;\frac{N-M_{1}}{2K_{2}}\leq M_{2}<\frac{N-M_{1}}{K_{2}}. \end{array}$$

For sub-regime 2.A, we assume $s_{1}=\left\lfloor \frac{K_{1}}{3}\right\rfloor$ and $s_{2}=K_{2}$. Using ⌊z⌋≥z/2 for any z≥1, we see that it is a valid choice of $s_{1},s_{2}$, since $K_{1}\geq 4$ and thus $\left\lfloor K_{1}/3\right\rfloor \geq 1$. Equating the values of $s_{1},s_{2}$ in (38), we obtain

(50) $$\begin{array}{ccc} R_{S_{1}}^{lb}\left(M_{1},M_{2}\right) & \geq & \frac{1}{N}\left[\left\lfloor \frac{K_{1}}{3}\right\rfloor K_{2}\left(N-\left\lfloor \frac{K_{1}}{3}\right\rfloor M_{1}-\left\lfloor \frac{K_{1}}{3}\right\rfloor K_{2}M_{2}\right)\right]\\ & \overset{\left(a\right)}{\geq } & \frac{1}{N}\left[\frac{K_{1}K_{2}}{6}\left(N-\frac{M_{1}K_{1}}{3}-\frac{M_{2}K_{1}K_{2}}{3}\right)\right]\\ & \overset{\left(b\right)}{\geq } & \frac{1}{N}\left[\frac{K_{1}K_{2}}{6}\left(N-\frac{N}{3}-\frac{N}{3}\right)\right]\\ & = & \frac{K_{1}K_{2}}{18}\\ & \geq & \frac{1}{18}\min\left\{K_{1}K_{2},\frac{M_{1}}{M_{1}+M_{2}K_{2}}\cdot \frac{K_{2}\left(N-M_{1}\right)}{M_{1}+\left(M_{2}-1\right)K_{2}}\right.\\ & & \left.+\frac{M_{2}K_{2}}{M_{1}+M_{2}K_{2}}\cdot \frac{\left(N-1\right)K_{2}M_{2}}{\left(M_{2}-1\right)\left(M_{1}+M_{2}K_{2}\right)}\right\}, \end{array}$$

where (a) follows from ⌊z⌋≥z/2 for any z≥1; and (b) follows from $M_{1}<N/K_{1}$, $M_{2}<N/\left(K_{1}K_{2}\right)$. Combining the result with (23a), we obtain

(51) $$R_{S_{1}}^{lb}\left(M_{1},M_{2}\right)\geq \frac{1}{18}R_{S_{1}}\left(\alpha^{*},\beta^{*}\right).$$

The remaining sub-regimes of this regime can be analyzed in a similar manner, thus we present only the values chosen for $s_{1}$ and $s_{2}$, as well as the final inequality for each sub-regime. The values $\left(\left\lfloor \frac{N}{3M_{2}K_{2}}\right\rfloor ,K_{2}\right)$, $\left(1,K_{2}\right)$, $\left(1,\left\lfloor \frac{N}{4M_{2}}\right\rfloor \right)$ and $\left(1,\left\lfloor \frac{N-M_{1}}{2M_{2}}\right\rfloor \right)$ are chosen for $\left(s_{1},s_{2}\right)$ in sub-regimes 2.B, 2.C, 2.D and 2.E, respectively. Moreover, inequalities (52) through (55) demonstrate the gaps for the same sub-regimes, respectively,

(52) $$\begin{array}{ccc} R_{S_{1}}^{lb}\left(M_{1},M_{2}\right) & \geq & \frac{2}{135}R_{S_{1}\left(\alpha^{*},\beta^{*}\right)}, \end{array}$$

(53) $$\begin{array}{ccc} R_{S_{1}}^{lb}\left(M_{1},M_{2}\right) & \geq & \frac{3}{64}R_{S_{1}}\left(\alpha^{*},\beta^{*}\right), \end{array}$$

(54) $$\begin{array}{ccc} R_{S_{1}}^{lb}\left(M_{1},M_{2}\right) & \geq & \frac{1}{32}R_{S_{1}}^{lb}\left(\alpha^{*},\beta^{*}\right), \end{array}$$

(55) $$\begin{array}{ccc} R_{S_{1}}^{lb}\left(M_{1},M_{2}\right) & \geq & \frac{1}{96}R_{S_{1}}\left(\alpha^{*},\beta^{*}\right). \end{array}$$

We will also study regime 3 through dividing it into two sub-regimes as follows:

$$\begin{array}{ccc} 3.A)\;\frac{N}{K_{1}}\leq M_{1}\leq N, & & \;\frac{N-M_{1}}{K_{2}}\leq M_{2}<\frac{N-M_{1}}{2},\\ 3.B)\;\frac{N}{K_{1}}\leq M_{1}\leq N, & & \;\frac{N-M_{1}}{2}\leq M_{2}\leq N. \end{array}$$

The reasoning method is similar to the case of sub-regimes 2.A through 2.E. Therefore, we briefly mention only the chosen values for $\left(s_{1},s_{2}\right)$ and the final inequality obtained for each sub-regime. For sub-regime 3.A, we chose $s_{1}=1$ and $s_{2}=\left\lfloor \frac{N-M_{1}}{2M_{2}}\right\rfloor$ and derive

(56) $$R_{S_{1}}^{lb}\left(M_{1},M_{2}\right)\geq \frac{1}{16}R_{S_{1}}\left(\alpha^{*},\beta^{*}\right).$$

In sub-regime 3.B, we obtain

(57) $$\begin{array}{ccc} R_{S_{1}}^{lb}\left(M_{1},M_{2}\right) & \geq & 0=\frac{8}{3}-\frac{8}{3}\\ & \geq & \frac{4}{3}\cdot 2\cdot 1-\frac{8}{3}\\ & \overset{\left(a\right)}{\geq } & \frac{K_{1}}{K_{1}-1}\cdot \frac{N-M_{1}}{M_{2}}\cdot \frac{N-M_{1}}{N}-\frac{8}{3}\\ & \geq & \frac{K_{1}}{K_{1}-1}\frac{{\left(N-M_{1}\right)}^{2}}{M_{2}N}-\frac{8}{3}\\ & = & \frac{M_{2}-1}{M_{2}}\cdot \frac{K_{1}}{K_{1}-1}\frac{{\left(N-M_{1}\right)}^{2}}{N(M_{2}-1)}-\frac{8}{3}\\ & \overset{\left(b\right)}{\geq } & \frac{5}{6}R_{S_{1}}\left(\alpha^{*},\beta^{*}\right)-\frac{8}{3}, \end{array}$$

where (a) follows from $\frac{N-M_{1}}{M_{2}}\leq 2$ and (b) follows from $N\geq K_{1}K_{2}$, $K_{1}\geq 4$, and (24a).

The results obtained for sub-regimes 3.A and 3.B suggest that the gap analysis for regime 3 will be similar to the case of regime 1 and regime 2. On the other hand, we show that regimes 1, 2 and 3 cover the entire $\left(M_{1},M_{2}\right)$ plane. This helps us come into the conclusion that in each subregime $R_{S_{1}}\left(\alpha^{*},\beta^{*}\right)$ and $R_{S_{1}}^{lb}\left(M_{1},M_{2}\right)$ are within a constant multiplicative and additive gap. Therefore, the unified final result which we will obtain for all the studied regimes is

(58) $$R_{S_{1}}\geq R_{S_{1}}^{lb}\left(M_{1},M_{2}\right)\geq \frac{1}{96}R_{S_{1}}\left(\alpha^{*},\beta^{*}\right)-4.$$

□

### 5.2. $R_{S_{2}}\left(\alpha^{*},\beta^{*}\right)$ against $R_{S_{2}}^{lb}\left(M_{1},M_{2}\right)$

**Theorem** **4.** $R_{S_{2}}\left(\alpha^{*},\beta^{*}\right)$ is within a constant multiplicative and additive gap with $R_{S_{2}}^{lb}$ for every possible value of $\left(M_{1},M_{2}\right)$. Specifically,

$$R_{S_{2}}\geq R_{S_{2}}^{lb}\left(M_{1},M_{2}\right)\geq \frac{1}{45}R_{S_{2}}\left(\alpha^{*},\beta^{*}\right)-16.$$

**Proof.**  Let us focus on the case where $N\geq K_{1}K_{2}$, $K_{1}\geq 4$ and $K_{2}\geq 4$. Recall from (25b) that achievable secure rate $R_{S_{2}}\left(\alpha^{*},\beta^{*}\right)$ is upper bounded as

(59) $$R_{S_{2}}\left(\alpha^{*},\beta^{*}\right)\leq K_{1}\cdot min\left\{K_{2},\frac{N-1}{M_{2}-1}\right\}.$$

Furthermore, the lower bound on $R_{S_{2}}\left(\alpha^{*},\beta^{*}\right)$ can be obtained from (43) as

(60) $$R_{S_{2}}^{lb}\left(M_{1},M_{2}\right)=\max_{t\in \{1,2,\ldots ,K_{2}\}}\frac{t(N-tM_{2})}{N}.$$

In the rest of our discussion we will partition the $\left(M_{1},M_{2}\right)$ plane by distinguishing the following two cases:

$$\begin{array}{ccc} \left(1\right)\; & & 1\leq M_{2}\leq \frac{N}{4},\\ \left(2\right)\; & & \frac{N}{4}\leq M_{2}\leq N. \end{array}$$

We will examine the mentioned cases in order to improve the margin of the gap.

$\left(1\right)\;1\leq M_{2}\leq \frac{N}{K_{2}}$, let $t=\left\lfloor \frac{1}{3}\min\left\{K_{2},\frac{N}{M_{2}}\right\}\right\rfloor$ in (60). This is a valid choice since $K_{2}\geq 4$. Thus,

$$1\leq \left\lfloor \frac{1}{3}\min\left\{K_{2},\frac{N}{M_{2}}\right\}\right\rfloor \leq \frac{K_{2}}{3}.$$

By feeding the value of *t* into (60), it follows that

$$\begin{array}{ccc} R_{S_{2}}^{lb}\left(M_{1},M_{2}\right) & \geq & \frac{1}{N}\left[\left\lfloor \frac{1}{3}\min\left\{K_{2},\frac{N}{M_{2}}\right\}\right\rfloor \cdot \left(N-\left\lfloor \frac{1}{3}\min\left\{K_{2},\frac{N}{M_{2}}\right\}\right\rfloor M_{2}\right)\right]. \end{array}$$

Since ∀z≥1:⌊z⌋≥z/2, we can continue as follows,

$$\begin{array}{ccc} R_{S_{2}}^{lb}\left(M_{1},M_{2}\right) & \geq & \frac{1}{N}\left[\frac{1}{6}\min\left\{K_{2},\frac{N}{M_{2}}\right\}\left(N-\frac{N}{3}\right)\right]\\ & = & \frac{1}{9}\min\left\{K_{2},\frac{N}{M_{2}}\right\}. \end{array}$$

Because $N\geq K_{1}K_{2}$ and $K_{1}\geq 4$, we have

(61) $$\begin{array}{ccc} R_{S_{2}}^{lb}\left(M_{1},M_{2}\right) & \geq & \frac{1}{9}\cdot \frac{4}{5}\min\left\{K_{2},\frac{N-1}{M_{2}-1}\right\}\\ & = & \frac{4}{45}\min\left\{K_{2},\frac{N-1}{M_{2}-1}\right\}\\ & \geq & \frac{1}{45}\cdot K_{1}\cdot \min\left\{K_{2},\frac{N-1}{M_{2}-1}\right\}. \end{array}$$

From (61) and (25b) we obtain

(62) $$\begin{array}{c} R_{S_{2}}^{lb}\left(M_{1},M_{2}\right)\geq \frac{1}{45}R_{S_{2}}\left(\alpha^{*},\beta^{*}\right). \end{array}$$

(2) For $\frac{N}{4}\leq M_{2}\leq N$, it holds that

$$\begin{array}{ccc} R_{S_{2}}^{lb}\left(M_{1},M_{2}\right) & \geq & 0=K_{1}\frac{N}{M_{2}}-K_{1}\frac{N}{M_{2}}\\ & \geq & K_{1}\cdot \min\left\{K_{2},\frac{N}{M_{2}}\right\}-K_{1}\frac{N}{M_{2}}\\ & \overset{\left(K_{1}\geq 4\right)}{\geq } & K_{1}\cdot \frac{3}{4}\min\left\{K_{2},\frac{N-1}{M_{2}-1}\right\}-16. \end{array}$$

Therefore,

(63) $$R_{S_{2}}^{lb}\left(M_{1},M_{2}\right)\geq \frac{3}{4}R_{S_{2}}\left(\alpha^{*},\beta^{*}\right)-16.$$

The entire $\left(M_{1},M_{2}\right)$ plane is obviously covered by cases 1) and 2). Thus, $R_{S_{2}}\left(\alpha^{*},\beta^{*}\right)$ and $R_{S_{2}}^{lb}\left(M_{1},M_{2}\right)$ are shown to be embraced by constant additive and multiplicative curves as shown by (64) which is derived via combining (62) and (63),

(64) $$R_{S_{2}}\geq R_{S_{2}}^{lb}\left(M_{1},M_{2}\right)\geq \frac{1}{45}R_{S_{2}}\left(\alpha^{*},\beta^{*}\right)-16.$$

□

## 6. Conclusions and Further Works

In this paper, we further developed the system model of a coded caching scheme by simultaneously assuming a hierarchical network and adversaries tapping the shared links in peak time. We calculated the secure achievable rates of each link in the proposed scheme. The parameters considered in previously-proposed hierarchical scheme have been reconsidered here to obtain approximate minimum achievable rates. Furthermore, we calculated the lower bound on the feasible rates. We showed that the secure achievable rates are within a constant multiplicative and additive gap to the corresponding lower bounds. These results are similar to those obtained in the non-secure hierarchical scheme, but the cost of security appears in the form of larger constants. Our work can be continued by proposing and evaluating yet more complex system models. More complicated models can assume that the adversary has access to the shared links in the placement phase or allow the users to issue more than one request in the delivery phase.

## Data Availability

Not applicable.

## References

[1] Maddah-Ali M.A., Niesen U. (2014). Fundamental limits of caching. IEEE Trans. Inf. Theory.

[2] Sengupta A., Tandon R., Clancy T.C. Decentralized caching with secure delivery. Proceedings of the IEEE International Symposium on Information Theory.

[3] Sengupta A., Tandon R., Clancy T.C. (2015). Fundamental limits of caching with secure delivery. IEEE Trans. Inf. Forensics Secur..

[4] Karamchandani N., Niesen U., Maddah-Ali M.A., Diggavi S.N. (2016). Hierarchical coded caching. IEEE Trans. Inf. Theory.

[5] Bai B., Li W., Wang L., Zhang G. (2019). Coded caching in fog-ran: b-matching approach. IEEE Trans. Commun..

[6] Cao H., Yan Q., Tang X. (2019). Reducing search complexity of coded caching by shrinking search space. IEEE Commun. Lett..

[7] Kim G., Hong B., Choi W., Park H. (2018). Mds-coded caching leveraged by coordinated multi-point transmission. IEEE Commun. Lett..

[8] Luo T., Peleato B. (2018). The transfer load-i/o trade-off for coded caching. IEEE Commun. Lett..

[9] Zhang J., Lin X., Wang X. (2018). Coded caching under arbitrary popularity distributions. IEEE Trans. Inf. Theory.

[10] Cao Y., Tao M. (2019). Treating content delivery in multi-antenna coded caching as general message sets transmission: A dof region perspective. IEEE Trans. Wirel. Commun..

[11] Ngo K.-H., Yang S., Kobayashi M. (2018). Scalable content delivery with coded caching in multi-antenna fading channels. IEEE Trans. Wirel. Commun..

[12] Yang Q., Gündüz D. (2018). Coded caching and content delivery with heterogeneous distortion requirements. IEEE Trans. Inf. Theory.

[13] Pedersen J., Amat A.G., Andriyanova I., Brännström F. (2019). Optimizing mds coded caching in wireless networks with device-to-device communication. IEEE Trans. Wirel. Commun..

[14] Shariatpanahi S.P., Caire G., Khalaj B.H. (2019). Physical-layer schemes for wireless coded caching. IEEE Trans. Inf. Theory.

[15] Tang A., Roy S., Wang X. (2018). Coded caching for wireless backhaul networks with unequal link rates. IEEE Trans. Commun..

[16] Panigrahi B., Shailendra S., Rath H.K., Simha A. (2015). Universal caching model and markov-based cache analysis for information centric networks. Photonic Netw. Commun..

[17] Cheng M., Jiang J., Yan Q., Tang X. (2019). Constructions of coded caching schemes with flexible memory size. IEEE Trans. Commun..

[18] Shangguan C., Zhang Y., Ge G. (2018). Centralized coded caching schemes: A hypergraph theoretical approach. IEEE Trans. Inf. Theory.

[19] Vilardebó J.G. (2018). A novel centralized coded caching scheme with coded prefetching. IEEE J. Sel. Areas Commun..

[20] Wang J., Cheng M., Yan X., Tang Q. (2019). Placement delivery array design for coded caching scheme in d2d networks. IEEE Trans. Commun..

[21] Asghari S.M., Ouyang Y., Nayyar A., Avestimehr A.S. (2019). An approximation algorithm for optimal clique cover delivery in coded caching. IEEE Trans. Commun..

[22] Zheng L., Yan Q., Chen Q., Tang X. (2019). Delivery design for coded caching over wireless multicast networks. IEEE Access.

[23] Zhang K., Tian C. (2018). Fundamental limits of coded caching: From uncoded prefetching to coded prefetching. IEEE J. Sel. Areas Commun..

[24] Bayat M., Mungara R.K., Caire G. (2019). Achieving spatial scalability for coded caching via coded multipoint multicasting. IEEE Trans. Wirel. Commun..

[25] Vettigli G., Ji M., Shanmugam K., Llorca G., Tulino A.M., Caire G. (2019). Efficient algorithms for coded multicasting in heterogeneous caching networks. Entropy.

[26] Zhong S., Wang X. (2018). Joint multicast and unicast beamforming for coded caching. IEEE Trans. Commun..

[27] Combes R., Ghorbel A., Kobayashi M., Yang S. (2018). Utility optimal scheduling for coded caching in general topologies. IEEE J. Sel. Areas Commun..

[28] Karat N.S., Thomas A., Rajan B.S. (2019). Error correction in coded caching with symmetric batch prefetching. IEEE Trans. Commun..

[29] Pääkkönen G., Barreal A., Hollanti C., Tirkkonen O. (2019). Coded caching clusters with device-to-device communications. IEEE Trans. Mob. Comput..

[30] Ibrahim A.A., Zewail A.M., Yener A. (2019). Coded caching for heterogeneous systems: An optimization perspective. IEEE Trans. Commun..

[31] Zhang J., Lin X., Wang C.-C., Wang X. Coded caching for files with distinct file sizes. Proceedings of the 2015 IEEE International Symposium on Information Theory (ISIT).

[32] Lampiris E., Elia P. (2018). Adding transmitters dramatically boosts coded-caching gains for finite file sizes. IEEE J. Sel. Areas Commun..

[33] Niesen U., Maddah-Ali M.A. (2014). Coded caching with nonuniform demands. IEEE Trans. Inf. Theory.

[34] Ding Y., Wang L., Wu H., Shen H.V., Poor X. Tradeoff of content sharing efficiency and secure transmission in coded caching systems. Proceedings of the IEEE International Conference on Communications (ICC).

[35] Kiskani M.K., Sadjadpour H.R. Secure coded caching in wireless ad hoc networks. Proceedings of the International Conference on Computing, Networking and Communications (ICNC).

[36] Zewail A.A., Yener A. Coded caching for resolvable networks with security requirements. Proceedings of the IEEE Conference on Communications and Network Security (CNS): The Workshop on Physical-Layer Methods for Wireless Security.

[37] Kamel M., Wigger S., Sarkiss M. Decentralized coded caching for wiretap broadcast channels. Proceedings of the IEEE Global Communications Conference (GLOBECOM).

[38] Suthan I., Chugh C.H.H., Krishnan P. An improved secretive coded caching scheme exploiting common demands. Proceedings of the IEEE Information Theory Workshop (ITW).

[39] Hachem J., Karamchandani N., Diggavi S.N. (2017). Coded caching for multi-level popularity and access. IEEE Trans. Inf. Theory.

[40] Lim S.H., Wang C., Gastpar M. (2017). Information-theoretic caching: The multi-user case. IEEE Trans. Inf. Theory.

[41] Sengupta A., Tandom R., Clancy T.C. Improved approximation of storage-rate tradeoff for caching via new outer bounds. Proceedings of the 2015 IEEE International Symposium on Information Theory (ISIT).

[42] Vijit K.K.P., Rai B.K., Jacob T. Towards the exact rate memory tradeoff in coded caching. Proceedings of the National Conference on Communications (NCC).

[43] Wei Y., Ulukus S. Coded caching with multiple file requests. Proceedings of the 55th Annual Allerton Conference on Communication, Control and Computing (Allerton).

[44] Maddah-Ali M.A., Niesen U. (2015). Decentralized coded caching attains order-optimal memory-rate tradeoff. IEEE/ACM Trans. Netw. (TON).

[45] Wei Y., Ulukus S. Novel decentralized coded caching through coded prefetching. Proceedings of the 2017 IEEE Information Theory Workshop (ITW).

[46] Shannon C.E. (1949). Communication theory of secrecy systems. Bell Syst. Tech. J..

